# Linkage analysis and residual heterozygotes derived near isogenic lines reveals a novel protein quantitative trait loci from a *Glycine soja* accession

**DOI:** 10.3389/fpls.2022.938100

**Published:** 2022-07-29

**Authors:** Yia Yang, Thang C. La, Jason D. Gillman, Zhen Lyu, Trupti Joshi, Mariola Usovsky, Qijian Song, Andrew Scaboo

**Affiliations:** ^1^Division of Plant Science and Technology, University of Missouri, Columbia, MO, United States; ^2^Plant Genetics Research Unit, United States Department of Agriculture-Agricultural Research Service, Columbia, MO, United States; ^3^Department of Electrical Engineering and Computer Science, University of Missouri, Columbia, MO, United States; ^4^Department of Health Management and Informatics, MU Institute of Data Science and Informatics and Christopher S. Bond Life Science Center, University of Missouri, Columbia, MO, United States; ^5^Soybean Genomics and Improvement Laboratory, United States Department of Agriculture-Agricultural Research Service, Beltsville, MD, United States

**Keywords:** wild soybean (*Glycine soja Sieb. and Zucc.*), *Glycine soja*, seed protein, seed oil, QTL

## Abstract

Modern soybean [*Glycine max (L.) Merr*] cultivars have low overall genetic variation due to repeated bottleneck events that arose during domestication and from selection strategies typical of many soybean breeding programs. In both public and private soybean breeding programs, the introgression of wild soybean (*Glycine soja Siebold and Zucc.*) alleles is a viable option to increase genetic diversity and identify new sources for traits of value. The objectives of our study were to examine the genetic architecture responsible for seed protein and oil using a recombinant inbred line (RIL) population derived from hybridizing a *G. max* line (‘Osage’) with a *G. soja* accession (PI 593983). Linkage mapping identified a total of seven significant quantitative trait loci on chromosomes 14 and 20 for seed protein and on chromosome 8 for seed oil with LOD scores ranging from 5.3 to 31.7 for seed protein content and from 9.8 to 25.9 for seed oil content. We analyzed 3,015 single F_4:9_ soybean plants to develop two residual heterozygotes derived near isogenic lines (RHD-NIL) populations by targeting nine SNP markers from genotype-by-sequencing, which corresponded to two novel quantitative trait loci (QTL) derived from *G. soja*: one for a novel seed oil QTL on chromosome 8 and another for a novel protein QTL on chromosome 14. Single marker analysis and linkage analysis using 50 RHD-NILs validated the chromosome 14 protein QTL, and whole genome sequencing of RHD-NILs allowed us to reduce the QTL interval from ∼16.5 to ∼4.6 Mbp. We identified two genomic regions based on recombination events which had significant increases of 0.65 and 0.72% in seed protein content without a significant decrease in seed oil content. A new Kompetitive allele-specific polymerase chain reaction (KASP) assay, which will be useful for introgression of this trait into modern elite *G. max* cultivars, was developed in one region. Within the significantly associated genomic regions, a total of eight genes are considered as candidate genes, based on the presence of gene annotations associated with the protein or amino acid metabolism/movement. Our results provide better insights into utilizing wild soybean as a source of genetic diversity for soybean cultivar improvement utilizing native traits.

## Introduction

Soybean [*Glycine max (L.) Merr*] is one of the most valuable crops in the world due to the high protein and oil content of its seed, which has uses as feed for livestock, a good source of protein and oil for human health, and the oil can be used as a biofuel stock (Masuda and Goldsmith, 2009). In 2020, the world’s total soybean production was approximately 353 million metric tons (FAOSTAT, 2022^1^; accessed on 5/06/2022). The increased use of soybean meal in animal feed as a protein source has been a major driving force in soybean production (Dei, 2011). Fifty-three percent of soybean meal sold in the United States was used in feed for poultry, 29% for swine feed, 8% for aquaculture, 7% for other animals, 2% for dairy, <1% for cattle feed, and <1% for companion animals (USB, 2019). Soybean oil is primarily used for human consumption as cooking oil, mayonnaise, and salad dressing but can also be used in industrial processes such as cement, construction materials, electrical insulation, plastic, paint, mineral oils, and numerous applications (Hammond et al., 2005).

Soybean cultivars have relatively low genetic variation due to evolutionary and breeding events during domestication, the founder effect, and selection, which can create genetic bottlenecks that can decrease genetic diversity, alter allelic frequencies, increase linkage disequilibrium (LD), and eliminate rare alleles (Halliburton and Halliburton, 2004). Hyten et al. (2006) studied four populations and reported a decrease in nucleotide diversity (π) from 2.17 × 10^–3^ in wild soybeans (*Glycine soja Siebold and Zucc.)* to 1.47 × 10^–3^ in landraces, to 1.14 × 10^–3^ in North American ancestors, and to 1.11 × 10^–3^ in elite cultivars. Similar declines in nucleotide diversity levels, which collectively point to bottleneck effects during soybean domestication, have been reported in multiple studies (Li et al., 2014; Zhou et al., 2015; Valliyodan et al., 2016). Hyten et al. (2006) also reported that 50% of the genetic diversity and 81% of the rare alleles have been lost during domestication while 60% of the genes show significant changes in allelic frequencies. Wild soybean germplasm pools represent a potentially rich source of rare and novel alleles associated with important native traits; however, crossing *G. soja* with *G. max* often results in undesirable traits from *G. soja* present in direct progeny, such as late flowering, hard seed coat, prostrate growth habit, small seed size, pod shattering, and black color seeds (Carter et al., 2004; Liu et al., 2007). Many potentially desirable genes from *G. soja* are thought to be linked to undesirable traits, making breeding with *G. soja* both time and resource intensive (Carter et al., 2004). Rare alleles are often lost during domestication or due to founder events, and such alleles have largely been uncharacterized for traits utilized in soybean breeding and improvement research (Hyten et al., 2006). Previous studies with wild soybean populations derived from crosses between *G. soja* and *G. max* identified new potential genes, alleles, and quantitative trait loci (QTL) for diverse traits such as yield and maturity (Li et al., 2008), soybean cyst nematode (Zhang et al., 2017), seed yield (Concibido et al., 2003), linolenic acid content (Pantalone et al., 1997), and seed protein content (Fliege et al., 2022).

Soybean seeds were produced by typical soybean cultivars with an average of ∼40% seed protein content and of ∼20% seed oil content on a dry weight basis (Wilson, 2004). There is a well-characterized known inverse relationship between seed protein and oil content, which is believed to be due to (1) single genes, which have impacts on multiple traits (pleiotropy), (2) tightly linked genes with different effects on different components (Hymowitz et al., 1972; Chung et al., 2003; Leamy et al., 2017), and (3) energy cost partitioning between protein and oil structural components (Egli and Bruening, 2007). Breeding efforts for increased yield in soybean have caused seed protein content to decrease (Brzostowski et al., 2017). Rincker et al. (2014) reported positive increases in grain yield and seed oil and a net decrease in seed protein over 80 years (1925–2005) of breeding in soybean maturity groups II, III, and IV. A similar study focused on maturity groups V, VI, and VII from 1930 to 2010 reported a positive linear rate of grain yield and seed oil improvement and a negative linear decrease in seed protein content (Boehm et al., 2019). Previous studies reported negative phenotypic correlations between seed yield and seed protein content (Chung et al., 2003; Mello Filho et al., 2004; Warrington et al., 2015). Breeding for higher seed protein, seed oil, and yield in soybean germplasm can be difficult due to the negative correlation between seed protein and yield and seed protein and seed oil and positive correlations between seed oil and yield (Wilson, 2004; Rincker et al., 2014).

Currently, there are 252 and 327 QTLs associated with seed protein and seed oil content, respectively, as reported in the SoyBase database (Grant et al., 2010;^2^ accessed on 4/26/2022), many of which were discovered through biparental linkage analysis (Leamy et al., 2017). The first two major seed protein/oil QTLs were discovered on chromosome (Chr.) 15 and 20 (Diers et al., 1992) from a cross between the *G. soja* accession PI 468916, a high protein wild soybean from Liaoning, China, and the *G. max* line A8-3356022, a maturity group III experimental line from Iowa State University. The *G. soja* allele for the most significant marker from Chr20 and Chr15 had an increase in seed protein of 2.4 and 1.7%, respectively. These two QTLs were subsequently confirmed and have been named cqSeed protein-001 (Fasoula et al., 2004) and cqSeed protein-003 (Nichols et al., 2006) for Chr15 and Chr20, respectively. Most recently, cqSeed protein-003 was fine mapped and positionally cloned identifying *Glyma.20G85100* as the causal gene (Fliege et al., 2022). Patil et al. (2018) studied an interspecific mapping population, consisting of 188 F_7:8_ RIL, from a cross between the cultivar Williams 82 and a *G. soja* accession PI 483460B and identified five QTLs for seed protein content on Chr6, 8, 13, 19, and 20 and nine QTLs for seed oil content on Chr2, 7, 8, 9, 14, 15, 17, 19, and 20 by composite interval mapping using bin markers. Two significant seed protein loci were reported on Chr20 and one seed oil locus was identified on Chr5 using GWAS (Patil et al., 2018). A combination of linkage and GWAS analysis identified four significant single nucleotide polymorphism (SNP) loci regions distributed on Chr2, 6, 9, and 20 for seed protein and oil (Zhang et al., 2019). The QTL on Chr20 explained the highest proportion of the phenotypic variance (7.27–9.39) and additive effect (0.56–0.75). All the QTLs intervals reported either overlapped with or were close to, regions reported in previous studies (Diers et al., 1992; Tajuddin, 2005; Qi et al., 2011; Pathan et al., 2013; Patil et al., 2018; Seo et al., 2019). Warrington et al. (2015) studied the Benning × Danbaekkong population and identified QTLs for seed protein and amino acid on Chr14, 15, 17, and 20, and mapped Chr20 which explained 55% of the phenotypic variation and contains the Danbaekkong allele.

A novel seed protein QTL on Chr14 and seed oil QTL on Chr8 was detected in a previous study conducted by our group in 2018 (La, 2018) using a recombinant inbred line (RIL) population created by crossing Osage (Burton et al., 2012) × PI 593983. Here, we report on validation studies using two residual heterozygotes derived near isogenic line (RHD-NIL) populations derived from two lines of the original RIL mapping population. The overall objective of this study was to (1) validate a seed protein QTL on Chr14; (2) validate a seed oil QTL on Chr8; (3) validate the RHD-NIL as true near isogenic lines (NIL); and (4) reduce the initial QTL interval and utilize NILs to fine map QTL to permit candidate gene identification.

## Materials and methods

### Plant materials and field experiments

The original QTL mapping population started from a cross between Osage (Burton et al., 2012) [*Glycine max (L.) Merr*.] and PI593983 (*G. soja Sieb. and Zucc*.) in North Carolina in 2011. During the winter of 2011/2012, the F_1_ generation was grown at a USDA-ARS winter nursery in Isabela, Puerto Rico (coordinates: 18o30’N, 67o1’W; soil type: Coto clay). The F_2_ generation was grown in Columbia, MO, United States, during the summer of 2012, single F_2_ plants were selected, and the F_3_ seeds were harvested from each single plant separately to constitute unique populations derived from each F_2_ plant. During the summer of 2013, 338 F_3_ plants were grown and harvested individually in Columbia, MO, at the Bay Farm Research Facility. In 2014, 338 F_3:4_ inbred lines were grown at Bradford Research Center in Columbia, MO (coordinates: 38o59’N, 92o12’W; soil type: Mexico silt loam), in 3-meter rows, for each line, and one plant was randomly harvested from within each line and row. The F_4:5_ seeds were then sent to the winter nursery in Isabela, Puerto Rico, for seed increase. In 2016, 164 F_4:6_ RILs were planted at Greenley Memorial Research Center in Novelty, MO (coordinates: 40o01’N, 92o11’W; soil type: Putnam silt loam), and at the Hundley-Whaley Research Center in Albany, MO, United States (coordinates: 40o15’N, 94o19’W; soil type: Grundy silt loam). In 2017, the field experiment was conducted at Bradford Research Center in Columbia, MO (coordinates: 38o59’N, 92o12’W; soil type: Mexico silt loam), and at Greenley Memorial Research Center in Novelty, MO, United States. In all years and locations, the 164 RILs were planted in two-row plots. Plot dimensions were 2.44 m by 2.29 m. Seeds were sown at the rate of 41 seeds m^–1^. The RILs were planted in a randomized complete block design with two replications in all environments. All experiments were planted by using a four-row ALMACO cone planter with Kinze row units (ALMACO, Nevada, IA, United States) and four rows spaced at 0.76 m. The seed was harvested at R8 by an ALMACO SPC-40 plot (ALMACO, Inc. Nevada, IA, United States).

Our QTL mapping identified several genomic regions associated with seed protein and oil content and RILs that were heterozygous at the QTL intervals, which were selected to have single plants harvested. About 13 RILs were selected due to heterozygous status and were grown at Bay Farm Research Facility in, Columbia, MO, United States, in 2018. In total, 3,015 single plants were genotyped at nine QTL intervals to identify plants with homozygous versions of each allele at each QTL. This effort led to the selection of 121 near-isogenic lines (NIL) representing two QTLs. Due to a limited number of seeds, 121 F_9:10_ NILs, were grown with two replications as hill plots (1–8 seeds per hill plot) in the summer of 2019 at Bay Farm Research Facility, Columbia, MO, United States, and Lee Greenley Memorial Jr. Research Facility, Novelty, MO, United States. In the summer of 2020, 53 F_9:11_ NILs were grown as hill plots (25 seeds per plot) with two replications at Bay Farm Research Facility, Columbia, MO, and Lee Greenley Memorial Jr. Research Facility, Novelty, MO, United States.

### Protein and oil analysis

For the 2016 and 2017 field trials, approximately 5 g of ground soybean seed was used to calculate reflectance spectra by using XDS-NIRS Rapid Content™. Analyzer (FOSS Analytical, Slangerupgade, Denmark) and ISIscan™ software. The spectra were used to calculate the contents of seed protein and oil using the equations which were previously developed (Choung et al., 2001) based on the spectra from standard samples, calibration, and validation assessments. The calibration database includes soybeans from all over the United States and Canada. Samples were ground with a Foss Knifetec grinder (5-1-5 second burst). A certified 80% reflectance reference was used to create a reference standard. The performance test was carried out by running four segments ten times and compiling the spectra. For 2019 and 2020 field trials, approximately 20 ml of whole seeds were allocated from each field plot across all years and ground using a Perten laboratory Mill 3600 grinder (Perten Instruments, Hägersten, Sweden). Samples were analyzed for seed protein and oil content on a dry weight basis *via* near-infrared spectroscopy (NIRS) using a Perten model DA 7250 (Perten Instruments, Hägersten, Sweden). NIRS calibrations were originally developed and are updated every year by Perten Instruments and technical staff of the University of Minnesota as part of a national consortium.

### Genotyping-by-sequencing and linkage map creation for Osage × PI 593983 RIL population

Leaf tissue was collected from a single field replicate (pool of 5-10 plants per RIL) and DNA was isolated from ∼40 mg of lyophilized leaf tissue from a pool of 5-10 plants per RIL using the DNeasy Plant Mini kit (QIAGEN, Valencia, CA, United States), according to the manufacturer’s instructions. DNA samples were then submitted to the Institute for Genomic Diversity (IGD) at Cornell University, where genotyping by sequencing (GBS) libraries were created (Elshire et al., 2011) using ApeKI, DNA ligase, and appropriate Illumina adapters. IGD carried out all library construction, Illumina sequencing, read mapping, and SNP calling using TASSEL. BWA 0.7.8-r455 program (Li and Durbin, 2009) was used to map sequencing to the ‘Williams 82’ Wm82.a2.v1 reference sequence (Schmutz et al., 2010; Goodstein et al., 2011). The TASSEL 5.0 pipeline was used to call SNPs and allele frequencies, and SNPs were filtered to exclude those with > 80% missing data. The LinkImpute program (Money et al., 2015) with the settings of 30 high LD sites and 10 nearest neighbors was used to impute missing data. Finally, parental genotypes were assigned using the ABH genotype function in TASSEL. Only those SNPs for which a definitive parental origin could be assigned were used for downstream genetic map creation and QTL mapping. The ABHGenotypes function in R (Furuta et al., 2017) was then used to correct GBS-related genotyping errors using the correctUnderCalledHets and correctStretches functions (settings were maxhaplength = 3).

A linkage map was constructed using the software package ‘qtl’ (Broman et al., 2003; Broman and Sen, 2009) in RStudio (R Core Team, 2020) with 4,652 SNP. Genetic distances were estimated *via* the ‘est.map’ function with a genotyping error rate set at 0.01. Each chromosome with excessive map distances (>200 cM) was evaluated by manual removal of single markers *via* the ‘droponemarker’ and ‘est.map’ functions. In addition, chromosomes 3 and 13 were split into 3 and 2 sub-chromosomes, respectively. Each of the chromosomal marker orderings was evaluated *via* the ripple function, and no better marker order was identified than that present in the original Wm82.a2.v1 assembly.

The ‘qtl’ software package (Broman et al., 2003; Broman and Sen, 2009) was used for QTL analysis. To detect QTL, Expectation-Maximization (EM) algorithm was used (Xu et al., 2000; Sen et al., 2009). Analyses were carried out by using the composite interval mapping ‘cim’ procedure with a 10 cM window. The empirical logarithm of odds (LOD) thresholds were calculated at the 10% level of probability with 1000 permutations for protein and oil contents (Churchill and Doerge, 1994). The percentage of phenotypic variance explained by the significant QTL was determined by the ‘effectplot’ function. The effect of each QTL was determined by using the ‘effectplot’ function, following the ‘sim.geno’ function with 1000 draws and an error probability of 0.01. The confidence intervals for each significant QTL were presented as 1.5-LOD by using the ‘lodint’ function. A graphical presentation of detected QTL was drawn using MapChart 2.32 software (Voorrips, 2002).

### Illumina array-based genotyping, and whole genome resequencing of residual heterozygotes derived near isogenic lines

In 2018, 3,015 single F_4:9_ soybean plants were genotyped *via* a commercial vendor (AgriPlex Genomics, Cleveland, OH, United States) for 28 markers, five markers corresponding to the Chr14 protein QTL and four markers to the Chr8 oil QTL, *via* multiplexed Next-Gen PlexSeq™ from AgriPlex Genomics (AgriPlex Genomics, Cleveland, OH, United States). Leaf tissues were collected from every plant in a 2-ml tube and then lyophilized for 48 h before the samples were sent to AgriPlex. AgriPlex Genomic performed all library construction, Illumina sequencing, and genotype calling *via* an in-house software called PlexCall™. Due to an unfortunate event issue with sample processing, only 39% of data (1,175 lines) was usable. Genotypes were recorded with AA to indicate alleles from parent 1 ‘Osage,’ BB alleles from PI 593983, and HH alleles from heterozygous. After removing all missing data and errors, NILs were selected to cover all genotypic classes for the Chr14 protein QTL: A total of 61 NILs were selected to carry forward and had recombination events, with 10 NILs fully homozygous for each of the parental regions (‘Osage,’ AA; ‘PI 593983,’ BB). An additional two NILs heterozygous for the entire region (HH) on Chr14 were also selected. For the Chr8 QTL, a total of 66 genotypes were advanced; 46 NILs had recombination events within the Chr8 QTL region, 10 NILs each were advanced, which were fully homozygous for each of the parental alleles (‘Osage,’ AA; ‘PI 593983,’ BB), and lastly, 2 NILs (HH) that were fully heterozygous for the Chr8 QTL region were selected.

During the summer of 2019, 121 F_9:10_ RHD-NIL were grown in field trials, and young trifoliate leaves were collected from every plant in the hill plots and bulked per plot during the V5 growth stage. A modified Cetyl Trimethyl Ammonium Bromide (CTAB) method (Doyle and Doyle, 1987) was used to extract high-quality DNA suitable for genotyping analysis and whole-genome resequencing (WGR). DNA samples were then sent to the USDA-ARS Soybean Genomics and Improvement Laboratory, located in Beltsville, MD, United States where they were analyzed using the BARCSoySNP6K BeadChip Illumina genotyping array (Song et al., 2020). Alleles were called using the software GenomeStudio v2.0.5 (Illumina, San Diego, CA, United States). Genotypic data quality control was conducted in TASSEL version 5.0 (Bradbury et al., 2007) with adjusted parameters described by Heim and Gillman (2017) by removing markers greater than 80% heterozygous and removing RHD-NIL that have greater than 10% missing data. ABH parental calls were conducted in TASSEL version 5.0, where AA represents homozygote ‘Osage,’ BB represents homozygote PI 593983, and AB or H represents heterozygous. Genotypic data were extracted from TASSEL version 5.0 and imported into RStudio version 1.2.1335 (R Core Team, 2020). The package ‘ABHgenotypeR’ (Furuta et al., 2017) was used to impute missing genotypes and was error-corrected based on flanking alleles with the adjusted parameter of maxHapLength = 3 based on the study of Zhu et al. (2021), resulting in a final total of 2,966 makers.

DNA from 53 RHD-NIL samples were also submitted to a commercial vendor, GENEWIZ for short-read whole-genome sequencing at approximately 15 × coverage. The resulting FASTQ files were analyzed to identify genomic variation *via* PGen, a large-scale next-generation resequencing (NGS) data analysis of genomic variations workflow (Liu et al., 2016a). PGen was used to efficiently facilitate large-scale NGS data analysis of genomic variations, which is available in both a Linux version and a web-based implementation integrated within SoyKB (Joshi et al., 2014) and KBCommons (Zeng et al., 2019). *G. max* Williams 82 was used as the reference genome, specifically the Wm82.a2.v1 assembly (Schmutz et al., 2010) was used as the reference genome for mapping. The workflow starts by accepting paired-end or single-end fastq reads as input and performs data quality checks as the first step using FastQC (Andrews, 2010). Only the filtered high-quality reads are later aligned against the reference genome using BWA (Li et al., 2012). Picard Tools (Picard, 2018) was also used at this step to locate duplicate molecules and assign all reads into groups with the default parameters. After alignment, SNPs were called using the Haplotype caller algorithm from the Genome Analysis Toolkit (GATK) (McKenna et al., 2010). Filtering criteria were defined in the INFO field in the vcf file, where QD stands for quality by depth, FS is Fisher strand values, and MQ is the mapping quality of variants. Detected variants were then filtered using the criteria “QD < 26.0 || FS > 60.0 || MQ < 40.0” for SNPs and “QD < 26.0 || FS > 200.0 || MQ < 40.0” for indels.

A total of 431,738 SNP were called on Chr14 from the whole genome resequencing (WGR) data. An adjusted strict quality control following Heim and Gillman (2017) were imposed in TASSEL version 5.0 to call parental genotypes. The minimum SNP count was set at 30, and SNP greater than 80% heterozygous and less than 10% allelic frequency were removed. SNPs were filtered again with the minimum SNP count at 35 out of 55 sequences, a maximum allelic frequency of 90%, and a minimum allelic frequency at 10%. The function ‘homozygous genotype’ was used to remove all heterozygous allele calls. The function ‘thin site by position’ was used to remove an SNP at every 2000 base pair. LD KNNi imputations were conducted and ABH parental calls were conducted in TASSEL version 5.0. Genotypic data were imported into RStudio version 1.2.1335, and the package ‘ABHgenotypeR’ (Furuta et al., 2017) was used for error correction using the adjusted parameter of maxHapLength = 5 based on the work form Zhu et al. (2021), resulting in 11,836 SNP markers.

### Linkage map creation for residual heterozygotes derived near isogenic lines

For the RHD-NIL population, the genetic map and QTL mapping for protein was created in RStudio version 1.2.1335 (R Core Team, 2020) using the package ‘qtl’ (Broman et al., 2003; Broman and Sen, 2009). There were 2,962 SNP6k markers across 20 chromosomes after dropping markers that were not present on more than 50 RHD-NILs. A total of 93 SNP6k markers were present on Chr14 and used for QTL mapping. The function ‘scanone’ and using the Expectation-Maximization (EM) algorithm (Dempster et al., 1977) and Haley–Knott regression method (Haley and Knott, 1992), which is the regression of the phenotypes on the multipoint QTL genotype probabilities, was used for interval mapping on the Chr14 protein QTL. A genetic map of the WGR SNP was created in RStudio version 1.2.1335 (R Core Team, 2020) using the package ‘qtl’ (Broman et al., 2003; Broman and Sen, 2009) for QTL mapping. The ‘findDupMarkers’ function identifies sets of markers that are in linkage or are genetically identical. A total of 11,836 SNPs were reduced to eight SNPs using the functions ‘findDupMarkers’ and ‘drop_markers’; these eight SNPs represent eight different regions as defined by recombination events. The ‘drop.markers’ keeps the first marker of the recombination regions and drops the remaining markers that are in linkage. QTL mapping was conducted using the function ‘cim’ for composite interval mapping on the Chr14 protein QTL with the number of marker covariates set at 5, a mapping interval of 10 centimorgan (cM), EM as the mapping method, and an error probability of 0.001. However, due to the low density of markers on Chr14, interval mapping was unable to narrow the QTL region.

### Statistical analysis

Statistical analysis was conducted in RStudio version 1.2.1335 (R Core Team, 2020) using the function ‘aov’ to compute the analysis of variance (ANOVA). Single marker analysis using the SNP called from the BARCSoySNP6K BeadChip genotyping array was used for validating the Chr8 oil QTL and Chr14 protein QTL. Genetic similarity was calculated in TASSEL version 5.0 using the ‘distance matrix’ function to validate the Chr14 RHD-NIL as true NIL. ANOVA and broad-sense heritability on an entry mean basis were calculated using phenotypic values of the two replicated lines in each environment. The ANOVA statistical model is shown below:


(1) $$y_{i\,j\,k}=\mu +G_{i}+G_{i}\,E_{j}+E_{j}+R_{k\,j}+e_{i\,j\,k}$$

where *y*_*ijk*_ represents the phenotype in the *i*th genotype under the *k*th environment being the *k*th replication within the *j*th environment, μ represents the population mean, *G_i_* represents the *i*th genotype, *G_i_E_j_* represents the *i*th genotype by *j*th environment interaction, *E_j_* represents the environmental effect, *R_k_* is the *k*th replication within the *j*th environment, and *e*_*ijk*_ represents the residual effects (Fehr et al., 1987; Bernardo, 2002). Broad-sense heritability on an entry-mean basis was estimated using the formula below:


(2) $$h^{2}=\frac{\sigma_{G}^{2}}{\sigma_{G}^{2}+\frac{\sigma_{G\,E}^{2}}{E}+\frac{\sigma_{e}^{2}}{R\,E}},$$

where *h*^2^ indicated broad-sense heritability on an entry-mean basis, $\sigma_{G}^{2}$ is the genotypic variance, $\sigma_{G\,E}^{2}$ is the genotype × environment variance, *E* is the number of environments, $\sigma_{e}^{2}$ is the error variance, and *R* is the number of replications (Falconer and Mackay, 1983; Fehr et al., 1987; Bernardo, 2002).

Significant differences between alleles for recombination regions were determined by using a modified Best Linear Unbiased Prediction mixed-linear model (Bernardo, 1994; Panter and Allen, 1995) in RStudio version 1.2.1335 (R Core Team, 2020) using the function ‘lmer’. The mixed-linear model is described below:


(3) $$y_{i\,k}=\mu +M\,1+M\,2+M\,3+M\,4+M\,5\;+M6+M\,7+M\,8+E_{j}+R_{k\,j}+e_{i\,k}$$

where μ is the mean, *M1* is the marker that represents the first recombination region, *M2* is the second recombination region, *M3* is the third recombination region, *M4* is the fourth recombination region, *M5* is the fifth recombination region, *M6* is sixth recombination region, *M7* is the seventh recombination region, *M8* is the eight recombination region, *E_j_* is the environmental effect, *R*_*kj*_ is the *k*th replication within the *j*th environment effect, and *e*_*ik*_ represents the residual effect. *M1* – *M8* are fixed effects and *E_j_* and *R*_*kj*_ are random effects (Bernardo, 1994; Panter and Allen, 1995).

For the 2016 and 2017 field trial data, the analysis of variance (ANOVA) was carried out by using PROC MIXED in SAS version 9.4 (SAS Institute Inc, 2002). Genotype was used as a fixed effect to test for significant genotypic differences among accessions for all traits. PROC CORR of SAS (SAS Institute Inc, 2002) was used to determine significance and correlation coefficients between oil and protein contents based on means of the RILs across replications and environments. PROC TTEST of SAS (SAS Institute Inc, 2002) was used to determine the differences between RILs with homozygous alleles from Osage and PI593983 at the same loci.

### Candidate genes selection

Gene models and gene annotations were extracted from SoyBase (Grant et al., 2010; accessed on 3/01/2021). Potential candidate genes were selected based on gene ontology (GO) biology descriptions, which were obtained from TAIR v 10 (03/27/14), and EuKaryotic Orthologous Groups (KOG) descriptions from Phytozome (Goodstein et al., 2011). Candidate genes were determined within regions based on the presence of GO terms for amino acid transportation, amino acid regulation, and amino acid biosynthesis.

### Molecular marker assay development

Recombination region 5 was used to develop Kompetitive allele-specific polymerase chain reaction (KASP) assay. Marker ED-5 (Gm14:8059955; Wm82.a2.v1; Figure 6 and Supplementary Table 1) was developed by KASP-by-design. The reaction mixture was prepared according to the standard protocol described by LGC Biosearch Technologies^3^. The fluorescent end-point genotyping assay was carried out using Roche LightCycler 480-II instrument (Roche Applied Sciences, Indianapolis, Indiana). DNA was extracted using a non-hazardous method that does not need chemicals, such as chloroform, bmercaptoethanol, and phenol (King et al., 2014).

**FIGURE 1:**
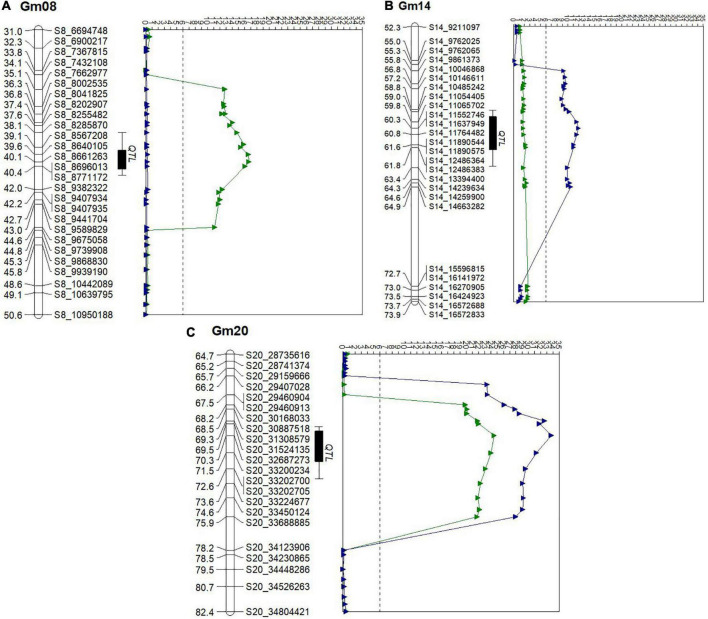
Quantitative trait loci (QTL) LOD score traces for seed oil (green) and protein (blue) content in a population of Osage × PI593983 across four environments during 2016 and 2017 in Missouri. The vertical axis indicates the genetic map position along the chromosome. The horizontal axis represents the logarithm of the odds (LOD) score. The black dotted line indicates the threshold of significance (LOD = 6.0). **(A)** Chr8. **(B)** Chr14. **(C)** Chr20.

**FIGURE 2:**
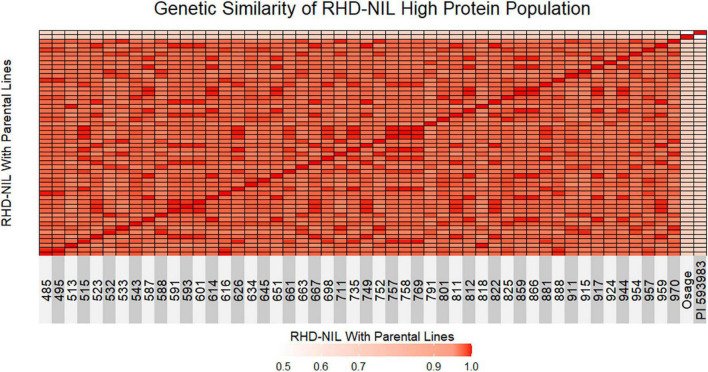
Genetic similarity test between individual RHD-NIL and parental lines is shown as a heatmap. Red indicates 1.0 genetically similar, light red indicates 0.90 genetically similar, and light pink indicates less than 0.50 genetically similar. Osage represents parent one and PI 593983 represents parent two.

**FIGURE 3:**
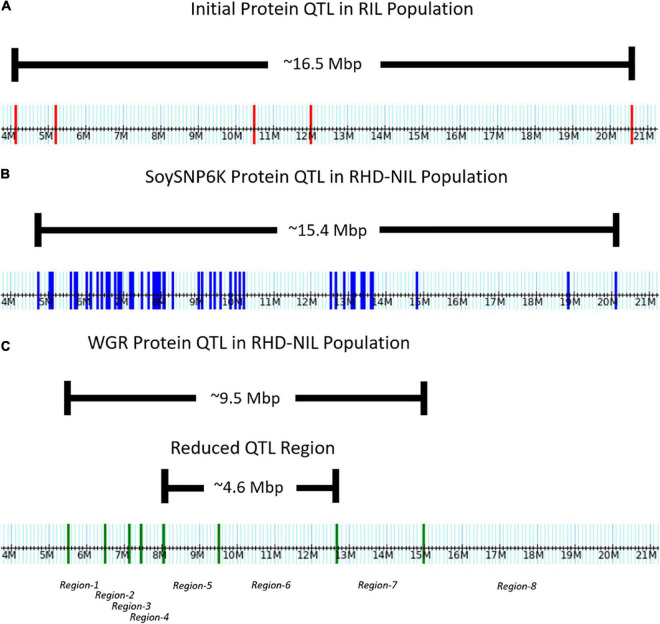
Distribution of markers across the Chr.14 protein QTL on the physical map. **(A)** Five genotyping-by-sequencing (GBS) markers in the initial RIL population. **(B)** Fifty-one SoySNP6K markers in the RHD-NIL population. **(C)** Eight WGR markers in the RHD-NIL population. The eight recombination regions are indicated on the physical map.

**FIGURE 4:**
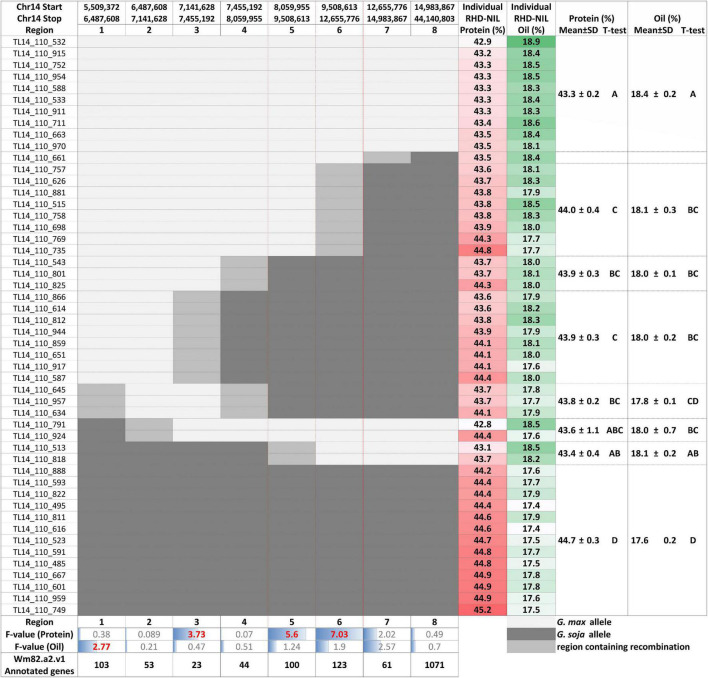
Individual RHD-NILs and their eight recombination regions with the physical start and stop positions, protein and oil content, and the mean with the standard deviation (SD) based on their *t*-test grouping. *F*-value for the protein and oil content and the number of Wm82.a2.v1 annotated genes are displayed at the bottom. *G. max* are white, *G. soja* are dark gray, and the region containing recombination is light gray. Regions 5 and 6 are outlined in red to showcase the most significant regions for seed protein content.

**FIGURE 5:**
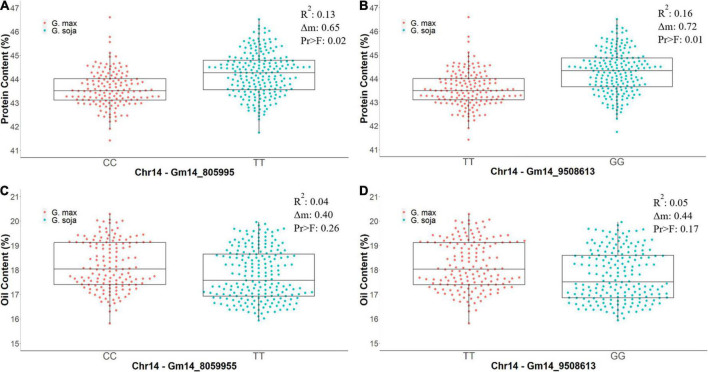
The differences in phenotypic values of protein content (%) and oil content (%) from CLM&NOV carrying different homozygous alleles for the markers Gm14_8059955 and Gm14_9508613. Allele (CC) is the allele from *G. max* (Osage) and (TT) is the allele from *G. soja* (PI 593983) in Gm14_8059955. The alleles in Gm14_9508613 are (TT) for *G. max* (Osage) and (GG) for *G. soja* (PI 593983). **(A)** Protein content for Gm14_8059955. **(B)** Protein content for Gm14_9508613. **(C)** Oil content for Gm14_8059955. **(D)** Oil content for Gm14_9508613. The whiskers represent the maximum and minimum values, the box displays the 25th and 75th percentile, and the line in the box is the median value. The dots represent the density of the phenotypic values.

**FIGURE 6:**
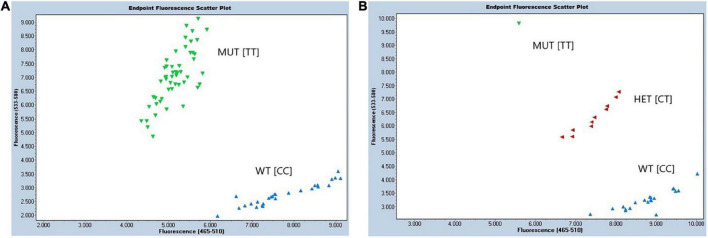
Performance of developed KASP assay ED-5 for detection of protein QTL on chr.14. Endpoint fluorescence scattering plots of **(A)** RHD-NIL population, and **(B)** BC_1_F_1_ breeding population. Allele-specific HEX primer (mutant; MUT) was displayed in green, allele-specific FAM primer (wild type; WT) was displayed in blue, and heterozygous (HET) lines were marked in red. The *X*-axis displays fluorescence of FAM at 523–568 nm, and the *Y*-axis displays fluorescence of HEX at 483–533 nm. Molecular marker assay for Gm14_8059955 is displayed above containing the forward FAM allele X wild type (WT), forward HEX allele Y mutant (MUT), and the reverse primer.

## Results

### Recombinant inbred line population phenotypic data

Soybean seed protein and seed oil contents were analyzed using data from four environments (16ALB, 16NOV, 17CLM, and 17NOV). Protein content ranged from 46.6 to 54.3%, with a mean of 50.8% across environments (Supplementary Table 2). Oil content ranged from 16.1 to 21.0% with a mean of 18.3% (Supplementary Table 2). The heritability based on the entry mean was 0.94 for seed protein and 0.92 for seed oil (Supplementary Table 2).

### Quantitative trait loci mapping on the recombinant inbred line population

A total of 548,086,161 reads were produced for 164 RILs, 8 *G. soja* lines including two parents of the RIL population and two blanks (no DNA controls), and 64.1% of the reads were found to map to single positions in the Williams 82 reference sequences (Schmutz et al., 2010; Goodstein et al., 2011). One RIL was determined to be unrelated by PCA analysis and was dropped from further analysis. SNPs were called in TASSEL and produced 170,463 raw SNPs and 139,012 filtered SNP positions, which had 6.687 and 7.019 mean site depth in the raw and filtered datasets, respectively. A total of two QTLs were detected on Chr8 and Chr20 with LOD values from 9.8 to 25.9 for seed oil content, respectively (Supplementary Table 3 and Figure 1). For seed protein content, three QTLs were identified on Chr14 and one QTL on Chr20 with LOD values ranging from 5.3 to 31.7. In this study, 27,248 markers were used to analyze polymorphism between parents Osage and PI593983 and among 164 RIL populations (Table 1). After removing markers for gap closure, the genetic linkage map covered 2,051.2 cM and included 4,374 markers on 20 chromosomes. Although more than 27,000 markers were used, >20,000 markers with distorted segregation were excluded (Table 1 and Supplementary Figure 1). This can be partly explained by the origin of the studied population, as the population was derived from a single F_2_ plant; approximately half of the genome would be fixed, leading to large gaps in the linkage map. Yet, this unique population structure also allows for fixing major effect QTL, such as the one widely reported on chromosome 15, which in turn allows for detection of smaller effect QTL.

**TABLE 1:** Genetic map distribution of GSB markers for the ‘Osage’ × PI 593983 RIL population.

	Number of Markers				Length (cM)	Average spacing (cM)	Max spacing (cM)
Chr	After imputation	Parental homozygous	Follow the rule of segregation	After removal for gap closure			
1	1,285	421	44	39	63.1	1.7	23.4
2	1,376	558	275	270	159.3	0.6	60.5
3A				365	8.4	0.4	1.8
3B	1,707	909	370	195	53.1	0.3	4.9
3C				146	47	0.3	5.1
4	1,464	557	264	210	74.5	0.4	8.5
5	1,198	486	231	216	111	0.5	20.1
6	1,249	392	165	156	57.2	0.4	3
7	1,207	351	143	137	95.5	0.7	24.3
8	1,565	650	241	237	143.9	0.6	67.6
9	1,209	368	14	10	61.5	6.8	31
10	1,219	362	180	177	113	0.6	34.2
11	1,357	614	294	253	123.9	0.5	16
12	1,329	685	331	312	102.3	0.3	6.4
13A	1,386	514	220	113	32.3	0.3	1.9
13B				82	29.7	0.4	2.5
14	1,973	1,245	546	522	177	0.3	7.8
15	981	182	55	56	57.8	1.1	19
16	1,387	693	314	284	123.4	0.4	6.8
17	1,110	394	221	212	75.8	0.4	3.2
18	1,391	397	178	166	99.9	0.6	59.5
19	883	75	27	22	67.9	3.2	50.5
20	1,972	1,172	539	535	173.6	0.3	6
Overall	27,248	11,025	4,652	4,374	2,051.2		

### Phenotypic analysis of the residual heterozygotes derived near isogenic lines

The phenotypic analysis for oil and protein content were conducted across five environments (18/19GH, 19CLM, 19NOV, 20CLM, and 20NOV), as well as a BLUP combining the two field seasons (CLM&NOV) and a BLUP for all environments, which includes the 2018/2019 greenhouse study (Combined). The 2018/2019 greenhouse study was ultimately left out of the CLM&NOV because the mean had too large of a margin under a mean-separation test to be grouped with the field studies, but the seed composition trends were concordant in terms of direction with field data. The greenhouse study’s oil content ranged from 20.1 to 22.1% and protein content ranged from 38.2 to 44.8 (Table 2). The oil content across environments ranged from 18.0 to 22.1%, while the combined field seasons ranged from 17.4 to 18.9% (Table 2). The coefficient of variation (CV) for oil content ranged from 1.82 to 3.10% across all environments. Protein content ranged from 38.2 to 46.6% across all environments, with the combined field environments ranging from 42.8 to 45.2% (Table 2). Overall, oil and protein content follow a relatively continuous and normal distribution (Table 2).

**TABLE 2:** Descriptive statistic of minimum, maximum, means, standard deviation (SD), coefficient of variation (CV), skewness, kurtosis, and least-square means of seed oil and protein between environments.

Traits	Environment^a^	Min	Max	Mean	*SD*	CV (%)	Skewness	Kurtosis	Groups^d^
Oil	18/19GH	20.1	22.1	21.1	0.51	2.41	0.08	–0.80	a
	19CLM	17.8	19.8	18.9	0.45	2.41	–0.05	–0.27	b
	19NOV	17.8	20.3	19.0	0.59	3.10	0.16	–0.39	b
	20CLM	16.2	17.9	17.2	0.43	2.49	–0.32	–0.48	c
	20NOV	16.2	18.3	17.1	0.48	2.83	0.27	–0.45	c
	CLM&NOV*^b^*	17.4	18.9	18.0	0.36	1.98	0.18	–0.51	
	Combined*^c^*	18.0	19.5	18.6	0.34	1.82	0.27	–0.14	
———————————————————————————————————————————————————————————————————————————–									
Protein	18/19GH	38.2	44.8	41.4	1.47	3.55	–0.10	–0.12	d
	19CLM	42.8	45.7	44.1	0.83	1.87	0.23	–1.03	a
	19NOV	41.4	46.6	43.8	0.96	2.20	0.50	1.31	bc
	20CLM	42.9	45.2	44.0	0.60	1.36	–0.11	–0.80	ab
	20NOV	42.7	45.4	43.8	0.73	1.66	0.46	–0.53	c
	CLM&NOV*^b^*	42.8	45.2	44.0	0.60	1.36	0.23	–0.83	
	Combined*^c^*	43.0	44.9	43.4	0.69	1.59	0.11	–0.81	

^a^Five environments: 2018/2019 greenhouse (18/19GH), 2019 Columbia (19CLM), 2019 Novelty (19NOV), 2020 Columbia (20CLM), and 2020 Novelty (20NOV).

^b^Combined seed oil and protein content from four field environments (19CLM, 19NOV, 20CLM, and 20NOV).

^c^Combined seed oil and protein content from five environments (18/19GH, 19CLM, 19NOV, 20CLM, and 20NOV).

^d^Grouping of least square means.

ANOVA and broad-sense heritability tests were conducted on an entry-mean basis for the following environments: 19CLM, 19NOV, 20CLM, and 20NOV (Table 3). The genotypic variance explained for seed protein content was quite large at 2.27 and was significant at a *p*-value < 0.001, with environmental variance at 1.86. For oil content, the environmental variance was the highest at 480.91, followed by the genotype variance at 4.21. Genotype and environment were both significant at a *p*-value < 0.001 for seed oil content. The entry mean-based heritability (*h*^2^) for seed protein content was 0.72 and seed oil content was 0.69. The results from the ANOVA suggested that the genotypes from the RHD-NIL had a bigger impact on the level of seed protein content and that the environment had a bigger impact on the level of seed oil content. The Pearson correlation analysis was conducted for phenotypic values of the RHD-NIL in each environment (Table 4), and highly significant correlations existed between seed oil and seed protein content between environments (*P* < 0.001).

**TABLE 3:** Analysis of variance summary for seed protein and seed oil with heritability (*h*^2^) on an entry-mean basis.

Source of variance	Df	Mean Sq	*F*-value	Pr( > F)	Mean Sq	*F*-value	Pr( > F)
		———– Protein ———–			———— Oil —————		
Genotype (G)	49	2.27	4.91	9.99E-15***	0.81	4.21	3.23E-12***
Environment (E)	3	1.86	4.03	8.46E-03**	92.68	480.91	< 2.22E15***
Genotype × Environment (GxE)	139	0.64	1.38	2.40E-02*	0.25	1.28	6.61E-02
Replications in Environment	4	1.66	3.61	7.15E-03**	0.07	0.37	8.29E-01
Residual	161	0.46			0.19		
*h* ^2^		0.72			0.69		

*Indicates significance at the 0.05 level (P < 0.05).

**Indicates significance at the 0.01 level (P < 0.01).

***Indicates significance at the 0.001 level (P < 0.001).

**TABLE 4:** Pearson correlation coefficient between seed oil and protein in the high protein RHD-NIL population across multiple environments.

		18/19GH		19CLM		19NOV		20CLM		20NOV		CLM&NOV*^a^*		Combined*^b^*	
Environment	Trait	Oil	Protein	Oil	Protein	Oil	Protein	Oil	Protein	Oil	Protein	Oil	Protein	Oil	Protein
18/19GH	Oil	1													
	Protein	–0.75***	1												
19CLM	Oil	0.46**	–0.49**	1											
	Protein	–0.48**	0.45**	–0.77***	1										
19NOV	Oil	0.27^ns^	–0.38*	0.29^ns^	–0.29^ns^	1									
	Protein	–0.41**	0.48**	–0.28^ns^	0.38*	–0.78***	1								
20CLM	Oil	0.42**	–0.38*	0.55***	–0.39*	0.32*	–0.31*	1							
	Protein	–0.40**	0.54***	–0.53***	0.53***	–0.27^ns^	0.39*	–0.55***	1						
20NOV	Oil	0.57***	–0.58***	0.54***	–0.50**	0.25^ns^	–0.37*	0.39*	–0.70***	1					
	Protein	–0.51**	0.49**	–0.54***	0.60***	–0.22^ns^	0.36*	–0.34*	0.55***	–0.70***	1				
CLM&NOV*^a^*	Oil	0.58***	–0.62***	0.79***	–0.65***	0.68***	–0.63***	0.74***	–0.68***	0.73***	–0.60***	1			
	Protein	–0.59***	0.63***	–0.68***	0.81***	–0.55***	0.73***	–0.50**	0.75***	–0.70***	0.79***	–0.83***	1		
Combined*^b^*	Oil	0.74***	–0.66***	0.79***	–0.65***	0.51**	–0.52***	0.76***	–0.67***	0.74***	–0.60***	0.94***	–0.78***	1	
	Protein	–0.72***	0.83***	–0.69***	0.75***	–0.50**	0.66***	–0.52***	0.76***	–0.74***	0.76***	–0.83***	0.94***	–0.85***	1

The red color indicates highly correlated and the white color not correlated in the heatmap in the upper right corner.

^a^Combined data from four field study environments.

^b^Combined data from five environments.

*Indicates significance at the 0.05 level (*P* < 0.05).

**Indicates significance at the 0.01 level (*P* < 0.01).

***Indicates significance at the 0.001 level (*P* < 0.001).

### Validation of the Chr14 protein quantitative trait loci and the high protein residual heterozygotes derived near isogenic lines population

SoySNP6K marker data for the RHD-NIL were examined to validate and quantify the impacts of the two QTL, detected using the F_4_-derived RIL population (seed oil QTL on Chr8 and a seed protein QTL on Chr14). The Chr8 oil QTL was not validated, whereas the Chr14 protein QTL was validated using 93 SoySNP6K markers. These findings suggested that Chr8 oil QTL was detected as a false positive from the RIL mapping population study and was not continued for further analysis. While Chr14 protein QTL was validated and was moved forward for fine mapping.

The next step was to validate that the high-protein RHD-NILs are in fact true NILs. A genetic similarity test indicated that the RHD-NILs are genetically similar (Figure 2). Between individual RHD-NIL, the similarity ranged from 96 to 99% genetically, whereas the Osage and PI 593983 displayed ∼49% genetic similarity, and the similarity in individual RHD-NIL compared to the parental lines ranged from 71 to 73% for Osage and 68 to 71% for PI 593983. These results indicated that the parental lines are genetically distinct, and individual RHD-NIL are genetically similar, which validated our RHD-NIL function as true NILs. The seed protein QTL on Chr14 is one of a few genomic locations across 20 chromosomes that are still divergent.

### Fine-mapping the Chr14 protein quantitative trait loci

In the RIL population, the Chr14 protein QTL detected was approximately 16.5 million base pairs (Mbp) (Figure 3A). The Chr14 protein QTL in the RHD-NIL population was examined using SoySNP6K markers but due to the diffuse markers, only a limited number of recombination events were detected and, significant gaps present between markers Gm14_4728306 and Gm14_20110020 spanned a physical distance of approximately 15.4 Mbp (Figure 3B). A total 11,836 SNP from WGR data resulted in eight recombination regions: Gm14_5509372 to Gm14_6485179 (region 1), Gm14_6487608 to Gm14_7138691 (region 2), Gm14_7141628 to Gm14_7453099 (region 3), Gm14_7455192 to Gm14_8048870 (region 4), Gm14_8059955 to Gm14_9506311 (region 5), Gm14_9508613 to Gm14_12648760 (region 6), Gm14_12655776 to Gm14_14976378 (region 7), and Gm14_14976378 to Gm14_44140803 (region 8). These eight recombination regions reduced the QTL interval to approximately 4.6 Mbp (Figure 3). The genetic position of the eight seed protein and seed oil recombination regions are 0.00, 3.32, 5.46, 16.06, 23.41, 25.57, 36.20, and 37.26 centimorgan (cM), respectively (Table 5).

**TABLE 5:** The eight recombination regions for seed protein and oil on Chr. 14.

Trait	Chr^a^	Recomb. Region^b^	Marker Interval^c^	Position (cM)	*R*^2^ (%)^d^	*F*-value^e^
Protein content	14	Region-1	Gm14_5509372-Gm14_6485179	0.00	10.47	0.39
		Region-2	Gm14_6487608-Gm14_7138691	3.32	13.13	0.89
		**Region-3**	**Gm14_7141628-Gm14_7453099**	**5.46**	**16.43**	3.73*
		Region-4	Gm14_7455192-Gm14_8048870	16.06	14.65	0.07
		**Region-5**	**Gm14_8059955-Gm14_9506311**	**23.41**	**12.61**	5.60**
		**Region-6**	**Gm14_9508613-Gm14_12648760**	**25.57**	**16.16**	7.03***
		Region-7	Gm14_12655776-Gm14_14976378	36.20	17.99	2.02
		Region-8	Gm14_14976378-Gm14_44140803	37.26	16.75	0.49
Oil content	14	Region-1	Gm14_5509372-Gm14_6485179	0.00	3.56	2.77
		Region-2	Gm14_6487608-Gm14_7138691	3.32	2.89	0.21
		Region-3	Gm14_7141628-Gm14_7453099	5.46	2.94	0.47
		Region-4	Gm14_7455192-Gm14_8048870	16.06	2.82	0.51
		Region-5	Gm14_8059955-Gm14_9506311	23.41	3.60	1.24
		Region-6	Gm14_9508613-Gm14_12648760	25.57	4.51	1.93
		Region-7	Gm14_12655776-Gm14_14976378	36.20	4.81	2.57
		Region-8	Gm14_14976378-Gm14_44140803	37.26	4.23	0.70

^a^Chromosome number.

^b^Name of recombination regions for protein and oil.

^c^Marker interval of the recombination regions; Gm14 represents Chr 14 and the follow number represents the physical position.

^d^Variation explained for protien and oil (R^2^) in percentage.

^e^ANOVA F-value.

*Indicates significant at the 0.1 level (P < 0.1).

**Indicates significant at the 0.05 level (P < 0.05).

***Indicates significant at the 0.05 level (P < 0.01).

Three of the eight recombination regions were significantly associated with seed protein content (Table 5 and Figure 4). Regions 5 and 6 had *F*-values of 5.60 (*P* < 0.05) and 7.03 (*P* < 0.01), respectively. Region 3 was also significant (*P* < 0.1) but with a much lower *F*-value of 3.73. The phenotypic variance (*R*^2^) explained for protein content ranged from 10.47 to 17.99% with region 3 at 16.43%, region 5 at 12.61%, and region 6 at 16.16% (Table 5). While the phenotypic variance (R^2^) explained for seed oil content ranged from 2.82 to 4.81% (Table 5).

Individual RHD-NILs were grouped, and a t-test was performed to compare allelic effects for seed protein and oil content (Figure 4). Regions 5 and 6 were the most significant recombination regions. Eight RHD-NIL had regions containing recombination for region 6 with an average protein content of 44% and an average oil content of 18.1% (Figure 4). Two RHD-NILs had regions containing recombination for region 5 with an average protein and oil content of 43.4 and 18.1%, respectively (Figure 4). This analysis fine mapped the QTL to regions 5 and 6 for protein content, and it spans from 8,059,955_12,655,776 bp for the *G. max* ‘Williams 82’ Wm82.a2.v1 reference assembly.

Residual heterozygotes derived near isogenic lines with the *G. soja* allele (TT) for region 1 (Gm14_805995) had significantly lower seed oil content overall (0.40%, Figure 5C) and decreased oil content specific to the combined CLM&NOV analysis (0.44%) (Figure 5D). The difference in oil content between lines with the *G. max* and *G. soja* alleles in the greenhouse study for Gm14_805995 and Gm14_95086 was 0.37% (Supplementary Figure 2C) and 0.42%, respectively (Supplementary Figure 2D). The lines with alleles at Gm14_8059955 and Gm14_9508613 were not significantly different for seed oil content with *p*-values at 0.26 and 0.17, respectively (Figure 5). For protein content, lines with the *G. soja* allele also saw an increase at an average of 0.65% (Gm14_805995) and 1.75% (Gm14_9508613) (Supplementary Figures 2A,B).

### Candidate gene prediction

Candidate genes were identified from our RHD-NIL-defined QTL regions based on the presence of biological process GO terms and/or EuKaryotic Orthologous Groups (KOG) annotations associated with amino acid biosynthesis process, amino acid regulations, and amino acid transportation retrieved from SoyBase^4^. A total of eight genes (Glyma.Wm82.a2.v1), four within region 5 and, four genes within region 6 (9,508,613 –12,648,760 bp) are considered as potential candidate genes (Table 6).

**TABLE 6:** Candidate protein-related genes within regions 5 and 6.

Gmax 2.0 Gene IDs^a^	Start	Stop	Biological Process Descriptions	KOG Annotations^b^	Region
Glyma.14G090200	8218662	8222883	Amino acid transport	Amino acid transporter protein	Region-5
Glyma.14G096200	9006426	9009129	Amino acid transport	NA	Region-5
Glyma.14G096600	9045982	9049959	Amino acid transport	Beta-fructofuranosidase (invertase)	Region-5
Glyma.14G098100	9259148	9266365	Cellular modified amino acid biosynthesis	NA	Region-5
Glyma.14G102700	10194805	10198050	Aromatic amino acid family biosynthetic process	Chorismate mutase	Region-6
Glyma.14G104800	10748114	10751676	Regulation of amino acid import	NA	Region-6
Glyma.14G105200	10798891	10799849	Regulation of amino acid export	NA	Region-6
Glyma.14G105900	10916033	10919283	Amino acid transport	NA	Region-6

^a^Gene ID based on Wm82.a2.v1 assemblies.

^b^EuKaryotic Orthologous Groups (KOG) gene descriptions.

### Kompetitive allele-specific polymerase chain reaction assay development

Diagnostic molecular KASP assay ED-5 has been developed to capture the protein QTL on Chr14 in high throughput back and forward crossing. Two assays ED-5 and ED-6 were developed for upstream SNP in region 5 (Gm14_8059955) and region 6 (Gm14_9508613), respectively. Assay ED-6 did not show correct clustering of both alleles when compared with ED-5. For ED-5, TT allele corresponded to the *G. soja* mutant (MUT), while CC allele corresponded to the *G. max* (WT) (Figure 6). The sequences of FAM- and VIC-labeled primers and a common reverse primer were summarized in Supplementary Data. This assay was run on the 121 F_9:10_ RHD-NIL population and was able to predict a phenotype in 79.8% (data not shown). Moreover, the ED-5 assay was validated as useful in selecting a BC_1_F_1_ population (Figure 6).

## Discussion

Multiple seed protein and oil QTL have been detected and studied on Chr5 (Pathan et al., 2013), Chr15 (Diers et al., 1992; Fasoula et al., 2004; Pathan et al., 2013; Warrington et al., 2015), and Chr20 (Diers et al., 1992; Nichols et al., 2006; Patil et al., 2018). Warrington et al. (2015) identified a protein QTL on Chr14 with a phenotypic variance of 5% derived from Benning × Danbaekkong. Zhang et al. (2004) identified a QTL on Chr14 from a Kefong No.1 × Nanong 113;8-2 and had a phenotypic variance of 12.4%. Many of the detected protein QTL on Chr14 have alleles derived from Asian landraces (Zhang et al., 2004; Warrington et al., 2015; Huang et al., 2020).

In the RIL population, the mean protein content was 50.8%, and the RHD-NIL population’s mean protein content was 44.9%. The average protein content of the RIL and RHD-NIL was much higher than the typical protein content in soybean of 40% (Liu, 1997; Wilson, 2004) and RIL averaged protein content greater than a collection of 600 wild soybean accessions of 48% (Leamy et al., 2017). The average oil content of the RIL and RHD-NIL population in this study was 18.3 and 19.5%, respectively. Both populations’ means for oil content were lower than the typical oil content of 20% (Liu, 1997; Wilson, 2004) and higher than the average oil content of 11% in wild soybean collection (Leamy et al., 2017). The heritability for protein and oil content in the RIL population was 0.94 and 0.92, respectively. While the heritability for the RHD-NIL population for protein was 0.72 and for oil content was 0.69. Both populations’ heritability was higher than the heritability for protein and oil at 0.54 and 0.66 (Panthee et al., 2005), respectively. Diers et al. (1992) reported similar heritability for oil at 0.92 and protein at 0.74. A core collection evaluation of *G. max* and *G. soja* seed composition reported 36–40% for *G. max* check lines and 39–48% for *G. soja* accessions for protein concentration and a variation of 21–25% for *G. max* check lines and 15–17% for *G. soja* accessions for oil concentration (La et al., 2019). Based on this and other studies, *G. soja* accessions tend to have more protein content and less oil content than *G. max* lines.

Our RIL population was developed from a single F_2_ plant of the cross between Osage and PI593983, and a genotype-by-sequencing approach was used to make a linkage map with 4,374 polymorphic SNP markers. This allowed us to identify a total of four QTL for seed protein and two QTL for seed oil.

In fact, for biparental RIL populations, the limited number of recombination events suggests that it is unnecessary to genotype lines with many markers (Song et al., 2020). Existing BeadChip array, such as the BARCSoySNP6K BeadChip Illumina genotyping array (Song et al., 2020), which is a subset derived from the BARCSoySNP50K BeadChip Illumina genotyping array (Song et al., 2013), has been shown to be a strong genetic research tool and has been used to identify QTL and genes associated with phenotypic traits like growth period (Liu et al., 2016b), seed oil and fatty acids content (Priolli et al., 2019), seed protein content (Nascimento et al., 2018), and see yield (Ye et al., 2018).

In this study, we leveraged two genotyping technologies (SoySNP6K and WGR data) along with phenotypic data collection to validate and fine map QTL using an RHD-NIL population. In our study, due to genotyping error in 2018, our total sample size greatly decreased, which then affected the number of recombination events in our RHD-NIL population. This caused the SoySNP6K markers to not efficiently fine map the Chr14 protein QTL due to limited genetic diversity and insufficient polymorphic markers. Therefore, we utilized WGR to sequence individual RHD-NIL, which enabled massive SNP calling. The advancement and lower cost of next-generation sequencing have become a strong tool in the field of genomics by allowing researchers to sequence whole genomes (Koboldt et al., 2013). Individual-based WGR obtains high-quality individual genotypes, which requires a high read depth to accurately identify SNP, short INDEL, and genotype calling (Nagasaki et al., 2015). NGS technology can generate thousands to millions of DNA sequences, which can be leveraged to define genomic regions, increase SNP density, and even identify molecular genetic causes for traits of interest (Park and Kim, 2016; Schaid et al., 2018). As NGS continues to advance and the cost continues to lower, researchers will be able to utilize this genomic tool for linkage analysis, fine mapping, gene cloning, and other scientific projects.

In our study, the seed protein QTL on Chr14 was validated by detecting an association between SoySNP6K markers with seed protein and oil content. However, the seed oil QTL we originally detected on Chr8 was found to be false positive by single marker analysis using SoySNP6K markers. We subsequently determined that the Chr8 oil QTL overlaps with the seed coat color inhibitor locus (*I* locus), which controls the production and accumulation of anthocyanin over the seed coat *via* posttranscriptional gene silencing (PTGS) triggered by double-stranded RNA (dsRNA) (Senda et al., 2012). It is located in a region harboring a cluster of inverted repeats of three chalcone synthase genes CHS1–CHS3–CHS4 on Chr8 (Clough et al., 2004). Indirect inferences for NIRS methods mean that sometimes large spectral differences (such as the confounding effect of black vs. yellow seedcoat coloration) can result in artefactual QTL mapping results.

Near-isogenic lines are the ideal populations to confirm QTL and to initiate fine-level genetic mapping because confounding effects from other genomic regions are removed, which allows one to accurately model the effect of the QTL. By examining multiple NILs, it is possible to break up a large QTL interval into much smaller intervals (Fridman et al., 2000; Jander et al., 2002; Song et al., 2015). In our study, we were able to decrease the size of the initial QTL detected in the RIL population considerably. Although we identified a very large number of polymorphisms (11,836 in total), limited recombination condensed these polymorphisms to a single representative marker per recombination region used for regression analysis. We were able to reduce the Chr14 protein QTL to two of the eight recombination regions (regions 5 and 6) that are significantly associated with the increase in seed protein content. Similar fine-mapping approaches have been conducted using either single marker regression or haplotype analysis. Haplotype analysis between every two markers and regression analysis of the haplotypes to the phenotypic data was performed to fine map a major flowering time QTL (Zhang et al., 2012). Recently, *Glyma.20G085100* (Gm20:31774770-31779804; Wm82.a2.v1) has been fine-mapped and cloned as a causative gene at a seed protein QTL on soybean chromosome 20, known as cqSeed Protein-003. This gene encodes a CCT motif protein of unknown function, but it is closely related to the soybean plant’s circadian machinery genes (Fliege et al., 2022). QTLs for seed oil (cqSeed Oil-004), seed yield (cqSeed Yield-001), and seed mass (cqSeed weight-003) are frequently identified in the same genetic region likely because of pleiotropy (Nichols et al., 2006).

In our study, the allele responsible for increased seed protein content is derived from a *G. soja* accession PI 593983. In multiple studied populations, the genetic diversity in *G. soja* is more diverse when compared to Asian landraces and North American germplasms (Hyten et al., 2006). The phenotypic variation explained for protein content in our study was 12.61% for region 5 and 16.16% for region 6. The *G. soja* allele (TT) on Gm_14_805995 had an increase of 0.65% in protein content, and the *G. soja* allele (GG) on Gm_14_9508613 had an increase of 0.72%. Both alleles were significant for protein content increase and both alleles were insignificant for changes in oil content. It is well known that soybean overall seed protein and seed oil are negatively correlated (Liu, 1997; Wilson, 2004). This QTL is intriguing because it may be unique in increasing seed protein content without a negative impact on seed oil content. This finding was observed in the RIL population and confirmed in our RHD-NIL population.

Whole-genome resequencing SNP can be translated into functional markers and allows for further research on haplotype and SNP variation using WGR data (Patil et al., 2016). Our study reduced the Chr14 protein QTL interval for predictive gene identification and to create a real-time polymerase chain reaction (RT-PCR) marker assay for breeding purposes. In this study, a total of eight protein candidate genes were identified, which are located in the physical interval of 8,059,955 to 12,648,760 bp. These candidate genes were selected based on their gene ontology annotations from SoyBase (Grant et al., 2010; accessed on 3/01/2021) related to protein transport, amino acid transport, amino acid biosynthesis, and amino acid regulations. These reported eight genes can be considered as potential candidate genes for seed protein, but additional research is required to further narrow our candidate gene list to identify a causative polymorphism(s) within a specific gene(s). A KASP assay for region 5 was created for RT-PCR for marker-assisted selection (MAS). This marker assay will assist in genetic selection for the Chr14 protein QTL in our backcrossing population and future elite lines.

In summary, we detected a total of seven QTLs associated with seed protein and oil content using an RIL population. We leveraged advances in genotyping methods to enable rapid development of two RHD-NIL populations and leveraged WGR data to fine map a major effect of Chr14 protein QTL. The QTL window was narrowed from approximately 16.5 Mbp to approximately 4.6 Mbp. A total of eight candidate genes are intriguing targets for future studies. However, additional research is still needed to further narrow the candidate gene list and ultimately identify which of the tens of thousands of polymorphisms identified in this study are causative for an increase in seed protein content without an apparent decrease in seed oil content. A KASP assay developed by this research is publicly available and allows for the rapid introgression of this novel *G. soja* protein QTL into high-yielding elite cultivars.

## Data availability statement

The raw data supporting the conclusions of this article will be made available by the authors, without undue reservation.

## Author contributions

YY and TL conducted the field experiments and collected the phenotype data. YY performed the single marker analysis and BLUP mixed-linear model. TL conducted the QTL mapping, with assistance from JG. JG created the linkage map, helped in QTL mapping, and helped in BLUP mixed-linear model. ZL performed the bioinformatics for SNP calling. AS and JG conceived the idea of the study, designed the experiments, supervised the students, and acquired the funding. YY, JG, MU, and AS co-wrote the manuscript. YY, JG, MU, and TL provided the tables and figures. All authors contributed to the article and approved the submitted version.

## Footnotes

1
https://fao.org

2
https://soybase.org

3
http://www.biosearchtech.com

4
http://www.soybase.org

## Supplementary material

Supplementary Figure 1 Distribution of genotyping-by-sequencing derived Osage (blue), PI593983 (yellow), and segregating (green) regions on 20 chromosomes of the physical map. The marker was determined to be from Osage or PI593983 if the frequency of the major allele was > 0.70. The marker was determined to be segregating if the frequencies of the major allele were > 0.25 and < 0.70. Physical locations of markers were shown on the vertical axis in bp.

Supplementary Figure 2 The differences in phenotypic values of protein content (%) and oil content (%) from the greenhouse study carrying different homozygous alleles for the markers Gm14_8059955 and Gm14_9508613. Allele (CC) is the allele from G. max (Osage) and (TT) is the allele from G. soja (PI 593983) in Gm14_8059955. The alleles in Gm14_9508613 are (TT) for G. max (Osage) and (GG) for G. soja (PI 593983). **(A)** Protein content for Gm14_8059955. **(B)** Protein content for Gm14_9508613. **(C)** Oil content for Gm14_8059955. **(D)** Oil content for Gm14_9508613. The whiskers represent the maximum and minimum values, the box displays the 25th and 75th percentile, and the line in the box is the median value.
