# Supplementary material for: Linkage analysis and residual heterozygotes derived near isogenic lines reveals a novel protein quantitative trait loci from a Glycine soja accession
Source: Front Plant Sci. 2022 Jul 29;13:938100. doi: 10.3389/fpls.2022.938100 (PMC9372550; doi:10.3389/fpls.2022.938100)
Supplement: Supplementary file 1 [file Data_Sheet_1.docx]

**Supplementary Table 1 |** Kompetitive allele specific polymerase chain reaction marker region-5 assay.

| **Position** | **WT/MUT** | **Forward_FAM_Allele_X_WT** | **Forward_HEX_Allele_Y_MUT** | **Reverse_Common** |
| --- | --- | --- | --- | --- |
| Gm14_8059955 | C/T | TATTTTCTAAAATTCTATCAAGATGGCCG | TATTTTCTAAAATTCTATCAAGATGGCCA | CTTGCTTTTTGGTGTGAAGCTTATTGGTT |

**Supplementary Table 2 |** Descriptive statistics for protein and oil content of the RIL’s from the cross Osage × PI593983 across four environments in 2016 and 2017.

| traits | Range (%) | Mean (%) | *h^2^*^a^ | CV (%)^b^ | P-value | LSD^c^ |
| --- | --- | --- | --- | --- | --- | --- |
| Oil | 16.1-21.0 | 18.3 | 0.94 | 1.08 | <0.0001 | 4.70 |
| Protein | 46.6-54.3 | 50.8 | 0.92 | 1.49 | <0.0001 | 8.60 |

^a^Heritability on an entry mean basis.

^b^Coefficient of variation.

^c^Least significant difference with α = 0.05.

**Supplementary Table 3 |** Quantitative trait loci identified associated with seed oil and protein content using a ‘Osage’ × PI 593983 RIL population across four environments during 2016 and 2017 in Missouri.

| **Trait** | **Peak ^a^** | **Confidence Interval ^a^** | **Interval Size** | **Environment** | **LOD** |
| --- | --- | --- | --- | --- | --- |
| Oil | S8_7883923 (36.4) | S8_6694748 - S8_9382322 (31.0 – 42.0) | 2.69 Mbp | 16ALB | 9.8 |
|  | S8_8565390 (38.9) | S8_7367815 - S8_9675058 (33.8 – 44.6) | 2.31 Mbp | 16NOV | 13.5 |
|  | S8_8661263 (40.1) | S8_7432108 - S8_9739908 (34.1 – 44.8) | 2.31 Mbp | 17CLM | 15.9 |
|  | S8_8661263 (40.1) | S8_7662977 - S8_9868830 (35.1 – 45.3) | 2.21 Mbp | 17NOV | 19.6 |
|  | **S8_8661263 (40.1)** | **S8_7432108 - S8_9739908 (34.1 - 44.8)** | **2.31 Mbp** | **MEAN** | **16.7** |
|  |  |  |  |  |  |
|  | S20_32687273 (70.3) | S20_29407028 - S20_34123906 (66.2 – 78.2) | 4.72 Mbp | 16ALB | 18.3 |
|  | S20_32687273 (70.3) | S20_29407028 - S20_34123906 (66.2 - 78.2) | 4.72 Mbp | 16NOV | 25.9 |
|  | S20_33200234 (71.5) | S20_32687273 - S20_34526263 (70.3 – 80.7) | 1.84 Mbp | 17CLM | 19.7 |
|  | S20_32687273 (70.3) | S20_28414826 - S20_33450124 (64.2 – 74.6) | 5.04 Mbp | 17NOV | 18.4 |
|  | **S20_32687273 (70.3)** | **S20_29460913 - S20_34123906 (67.5 - 78.2)** | **4.66 Mbp** | **MEAN** | **24.5** |
|  |  |  |  |  |  |
| Protein | S14_11065702 (59.8) | S14_9861373 - S14_15596815 (55.8 – 72.7) | 5.74 Mbp | 16ALB | 10.5 |
|  | S14_14663282 (64.9) | S14_11065702 - S14_15596815 (59.8 – 72.7) | 4.53 Mbp | 16NOV | 9.3 |
|  | S14_28073066 (90.5) | S14_19596123 - S14_28073381 (79.2 – 91.1) | 8.48 Mbp | 17CLM | 5.3 |
|  | S14_42568158 (122.6) | S14_41258112 - S14_43747871 (116.9 – 128.4) | 2.49 Mbp | 17NOV | 8.2 |
|  | **S14_11637949 (60.3)** | **S14_9762065 - S14_15596815 (55.3 – 72.7)** | **5.83 Mbp** | **MEAN** | **12.0** |
|  |  |  |  |  |  |
|  | S20_32687273 (70.3) | S20_29407028 - S20_34123906 (66.2 - 78.2) | 4.72 Mbp | 16ALB | 27.8 |
|  | S20_32687273 (70.3) | S20_29407028 - S20_34123906 (66.2 - 78.2) | 4.72 Mbp | 16NOV | 31.7 |
|  | S20_32687273 (70.3) | S20_28852660 - S20_33622818 (65.2 – 75.4) | 4.77 Mbp | 17CLM | 21.6 |
|  | S20_25575883 (56.7) | S20_18790114 - S20_27727580 (51.6 – 62.4) | 8.94 Mbp | 17NOV | 20.4 |
|  | **S20_32687273 (70.3)** | **S20_29407028 - S20_34123906 (66.2 - 78.2)** | **4.72 Mbp** | **MEAN** | **33.8** |

^a^Peak and confidence interval markers correspond to physical SNP position in base pairs (bp) based on Wm82.a2.v1, and genetic position marked in parenthesis in centiMorgans (cM) based on the linkage map.
