# Supplementary figures and images for: Linkage analysis and residual heterozygotes derived near isogenic lines reveals a novel protein quantitative trait loci from a Glycine soja accession
Source: Front Plant Sci. 2022 Jul 29;13:938100. doi: 10.3389/fpls.2022.938100 (PMC9372550; doi:10.3389/fpls.2022.938100)

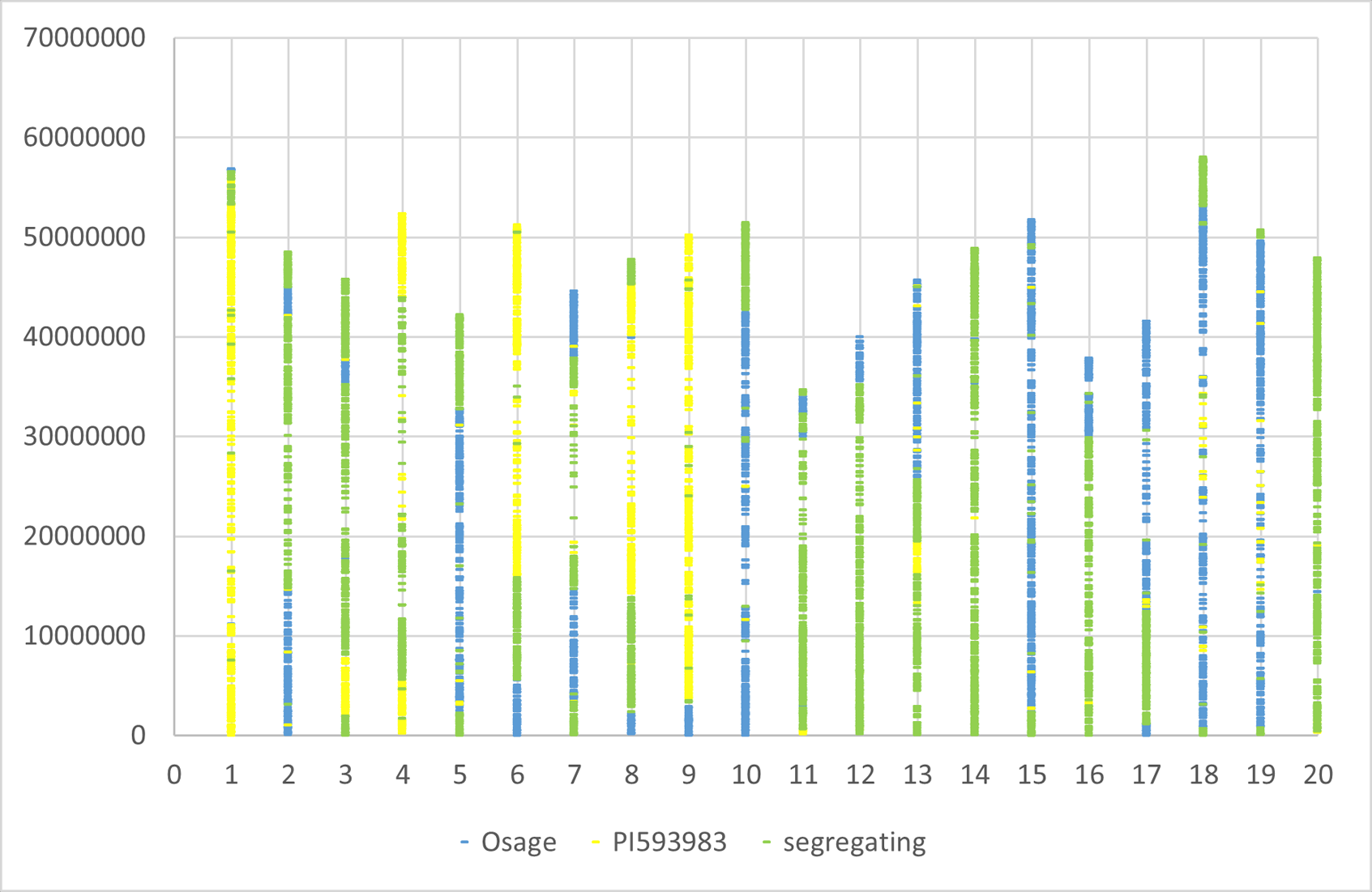

Supplement: Supplementary Figure 1 — Distribution of genotyping-by-sequencing derived Osage (blue), PI593983 (yellow), and segregating (green) regions on 20 chromosomes of the physical map. The marker was determined to be from Osage or PI593983 if the frequency of the major allele was > 0.70. The marker was determined to be segregating if the frequencies of the major allele were > 0.25 and < 0.70. Physical locations of markers were shown on the vertical axis in bp. [file Image_1.TIFF]

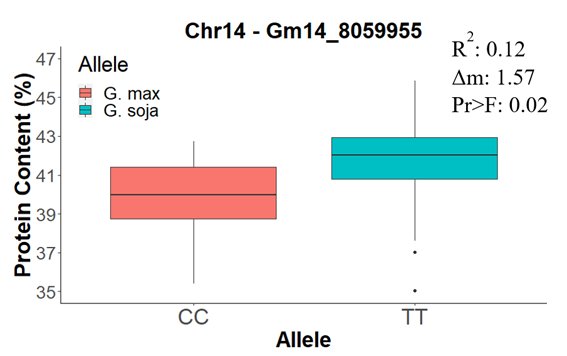

Supplement: Supplementary Figure 2 — The differences in phenotypic values of protein content (%) and oil content (%) from the greenhouse study carrying different homozygous alleles for the markers Gm14_8059955 and Gm14_9508613. Allele (CC) is the allele from G. max (Osage) and (TT) is the allele from G. soja (PI 593983) in Gm14_8059955. The alleles in Gm14_9508613 are (TT) for G. max (Osage) and (GG) for G. soja (PI 593983). (A) Protein content for Gm14_8059955. (B) Protein content for Gm14_9508613. (C) Oil content for Gm14_8059955. (D) Oil content for Gm14_9508613. The whiskers represent the maximum and minimum values, the box displays the 25th and 75th percentile, and the line in the box is the median value. [file Image_2.TIFF]

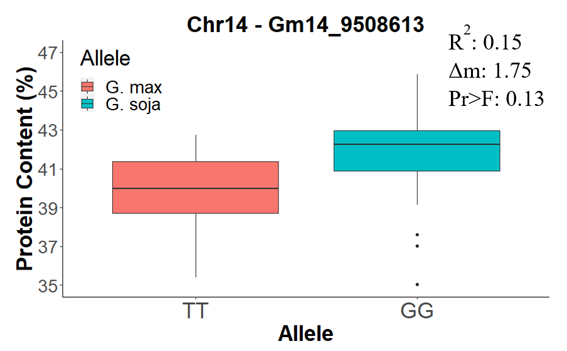

Supplement: Supplementary file 4 [file Image_3.TIFF]

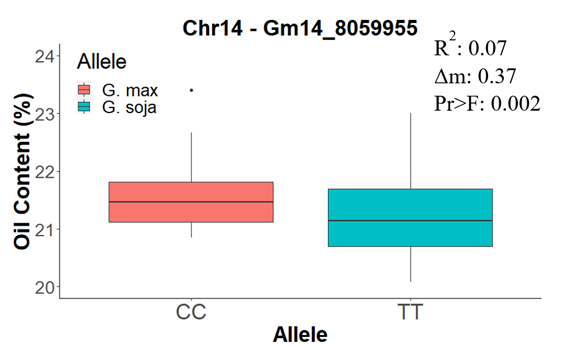

Supplement: Supplementary file 5 [file Image_4.TIFF]

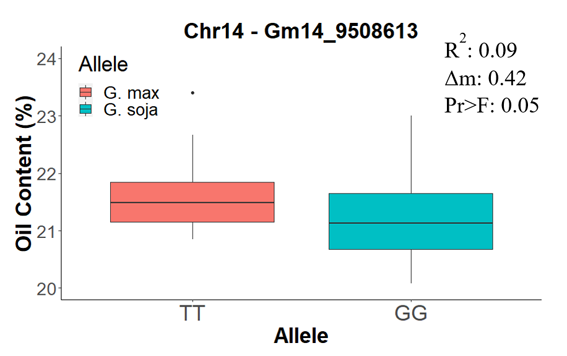

Supplement: Supplementary file 6 [file Image_5.TIFF]
